# The use of conceptual components in language production: an ERP study

**DOI:** 10.3389/fpsyg.2014.00363

**Published:** 2014-04-29

**Authors:** Alexandra Redmann, Ian FitzPatrick, Frauke Hellwig, Peter Indefrey

**Affiliations:** ^1^Abteilung für Allgemeine Sprachwissenschaft, Institut für Sprache und Information, Heinrich Heine Universität DüsseldorfDüsseldorf, Germany; ^2^Centre for Cognitive Neuroimaging, Donders Institute for Brain, Cognition, and Behavior, Radboud University NijmegenNijmegen, Netherlands

**Keywords:** language production, ERPs, conceptual representation, semantic priming, color-diagnosticity

## Abstract

According to frame-theory, concepts can be represented as structured frames that contain conceptual attributes (e.g., “color”) and their values (e.g., “red”). A particular color value can be seen as a core conceptual component for (*high color-diagnostic*; HCD) objects (e.g., bananas) which are strongly associated with a typical color, but less so for (*low color-diagnostic*; LCD) objects (e.g., bicycles) that exist in many different colors. To investigate whether the availability of a core conceptual component (color) affects lexical access in language production, we conducted two experiments on the naming of visually presented HCD and LCD objects. Experiment 1 showed that, when naming latencies were matched for colored HCD and LCD objects, achromatic HCD objects were named more slowly than achromatic LCD objects. In Experiment 2 we recorded ERPs while participants performed a picture-naming task, in which achromatic target pictures were either preceded by an appropriately colored box (primed condition) or a black and white checkerboard (unprimed condition). We focused on the P2 component, which has been shown to reflect difficulty of lexical access in language production. Results showed that HCD resulted in slower object-naming and a more pronounced P2. Priming also yielded a more positive P2 but did not result in an RT difference. ERP waveforms on the P1, P2 and N300 components showed a priming by color-diagnosticity interaction, the effect of color priming being stronger for HCD objects than for LCD objects. The effect of color-diagnosticity on the P2 component suggests that the slower naming of achromatic HCD objects is (at least in part) due to more difficult lexical retrieval. Hence, the color attribute seems to affect lexical retrieval in HCD words. The interaction between priming and color-diagnosticity indicates that priming with a feature hinders lexical access, especially if the feature is a core feature of the target object.

## Introduction

In order to communicate our thoughts to other human beings, it is crucial to be able to refer to concepts shared with the listener. These concepts are formed whenever a new object or event is encountered, and constantly revised in interaction with the environment. But how are concepts represented in the mind? Are they structurally complex, or represented as interconnected unitary nodes? What consequences do different models of representation have for lexical access? Different lines of theories have proposed answers to these questions. According to decompositional accounts of conceptual representation (e.g., Goldman, 1975; Jackendoff, 1990; Bierwisch and Schreuder, 1992), conceptual features (such as *female* and *parent*) are combined to access the concept (such as mother^1^). No direct representation of the whole concept (i.e., a single conceptual node for mother) is used to access the lemma. This view on conceptual representation has been adopted by some models of lexical access (e.g., Dell, 1986; Finkbeiner and Caramazza, 2006). According to non-decompositional accounts (e.g., Collins and Loftus, 1975; Roelofs, 1992, 1993, 1997; Levelt et al., 1999), on the other hand, concepts are represented as whole conceptual nodes, which are connected to other conceptual nodes representing features. In contrast to decompositional accounts, non-decompositional accounts propose a single abstract representation of the whole concept (e.g., a conceptual node mother), which in itself does not include features of the concept but is connected to conceptual nodes such as female and parent via conceptual relations. In terms of lexical access, non-decompositional accounts assert that each lemma node is connected to and activated by a single conceptual node. Note that decompositional and non-decompositional semantics often make the same predictions and are difficult to tease apart. A recent attempt to model the cumulative semantic inhibition effect showed no differences when either decompositional or non-decompositional semantics were assumed (Howard et al., 2006).

The question of (non-)decompositionality of lexical selection is important for theories that try to model conceptual representations as frames (e.g., Barsalou, 1992, 2008; Barsalou et al., 2003). Building upon earlier frame-based approaches (e.g., Fillmore, 1968; Minsky, 1977), Barsalou (1992) introduces frames as structured representations consisting of attribute-value sets linked to a central node (e.g., the attribute *wheels* with the value *four* in the frame of car), structural invariants (i.e., correlational relations between attributes, such as the spatial relation between *seat* and *back* in the frame of chair) and constraints (regulating interdependent sets of attributes within a frame, e.g., *speed* and *duration* in the transportation frame). The frame of the concept lemon, for example, comprises information about its shape, taste, surface texture and color, and about where and how it is cultivated. Some of this information might be activated whenever the concept is activated (core features), other information can be dependent on the linguistic context (peripheral features; Barsalou, 1982). Frame-theoretical accounts are appealing because of their ability to model relationships between conceptual features (which feature list-based approaches and non-decompositional network models do not). However, at present frame-theory is underspecified with regard to the issue of (non-)decompositionality in lexical access. Frame representations could function like non-decompositional accounts if only the central node were to be used for lexical access, or they could function like compositional accounts if all features contained in the frame contribute to lexical access. In the present study, our goal is to better understand the role of features for lexical access: Is the lemma node only linked to whole conceptual nodes (such as tomato), or also to single attributes (such as its shape, *round*, or its color, *red*)? By contrasting the predictions made by decompositional and non-decompositional models of language production in this manner, we hope to contribute to a further refinement of frame-theory.

### Color-diagnosticity

One example of a feature that could be activated both in a context dependent as well as a context independent manner is color. Color may, for instance, be a core feature for certain concepts (e.g., a banana or a tennis ball) and a more peripheral feature for others (e.g., a car or a butterfly; Rubio-Fernandez, 2008). The degree to which a concept is associated with a particular typical color has been called *color-diagnosticity* (Biederman and Ju, 1988), *high color-diagnostic* (HCD) objects being more strongly associated to a particular, typical color than *low color-diagnostic* (LCD) objects. A number of behavioral and neurophysiological studies suggest that color contributes to object recognition in various tasks, including picture-naming (e.g., Price and Humphreys, 1989; Wurm and Legge, 1993; Humphrey et al., 1994; Vernon and Lloyd-Jones, 2003; Therriault et al., 2009; Bramão et al., 2010, 2011b; for reviews see Tanaka et al., 2001; Bramão et al., 2011c). According to the *color-diagnosticity hypothesis* (Tanaka and Presnell, 1999; Nagai and Yokosawa, 2003), the facilitatory effect of color on object recognition and naming should only be present for HCD objects (i.e., objects with a strongly associated color), and not (or less so) for LCD objects (i.e., objects with no strongly associated color). In a classification task, Tanaka and Presnell (1999) presented two written nouns on the left and right side of the screen. After a period of 2500 ms, a HCD or LCD picture appeared in the center of the screen. Each picture was presented once as a color photograph and once as an achromatic photograph to each participant during the experiment. The participants then decided which of the two names on the screen matched the object in the picture. In this task, as well as in an additional picture-naming study in which participants named colored and achromatic pictures, the authors found shorter reaction times for colored versions of HCD pictures as compared to achromatic versions, but no significant difference between colored and achromatic versions of LCD pictures. Other studies, however, found that color seems to facilitate recognition of pictures in a number of tasks regardless of the color-diagnosticity of the depicted object (Rossion and Pourtois, 2004; Uttl et al., 2006; Bramão et al., 2010; see also the meta-analysis by Bramão et al., 2011c). To date, only a few studies have investigated the role of color and color-diagnosticity in lexical processing (e.g., Lu et al., 2010; Bramão et al., 2012). Some indications that color-diagnosticity might influence lexical processing come from a study using Event-Related Potentials (ERPs) by Bramão et al. (2012). They presented colored and achromatic HCD and LCD objects in a picture-naming task in which participants were asked to type the object's name after viewing the picture without pronouncing the object's name. Bramão et al. (2012) focused on the early visual components P1 and N1 and the later components N300 and N400. Their results showed a more pronounced P1 and N1 for achromatic compared to colored pictures irrespective of the color-diagnosticity of the target object. According to the authors, these results show that colored presentation places smaller demands on visual processing than achromatic presentation, possibly due to a facilitatory effect of color on shape segmentation processes. Further, they found an effect of color on the N400 for HCD objects but not for LCD objects, suggesting that color influences later processing stages in the naming of these objects. However, one limitation of the study by Bramão et al. (2012) is that they did not study overt speech production, but rather required their participants to type in the names of the stimuli after a short delay. As a consequence, it is difficult to judge whether their reported lexico-semantic effects of color-diagnosticity reflect true “online” speech production, or whether these effects could have been susceptible to more “offline” processes. Further, they presented the object and the color simultaneously (thereby temporally conflating the processing of the object and its color), making it hard to dissociate the effects of the visual processing of color and the processing of the color feature. Thus, the influence of color-diagnosticity on lexical processing has not been conclusively established. To do so, it would be necessary to directly investigate the time-course of color feature activation during overt speech production, ideally in a context that separates the processing of the color feature from the processing of the object.

### Electrophysiology of language production

A promising avenue for investigating the time-course of lexical processing in overt speech production is the recording of ERPs. In order to avoid speech-related motor artifacts, previous studies using ERPs mainly focused on tasks that did not require overt motor responses, for instance, covert or delayed naming (e.g., Schmitt et al., 2001; Jescheniak et al., 2002). Recently, however, a number of studies have shown that it is indeed possible to use EEG recordings in overt speech production tasks such as picture-naming (e.g., Eulitz et al., 2000; Christoffels et al., 2007; Chauncey et al., 2009; Costa et al., 2009; Strijkers et al., 2010, 2011; Laganaro and Perret, 2011).

With respect to language production, the P2 component has been shown to reflect difficulty of lexical access by Strijkers et al. (2010; see also Costa et al., 2009; Strijkers et al., 2011, 2013). Strijkers et al. (2010) presented Spanish-Catalan bilinguals with pictures of lexical items that were orthogonally manipulated for lexical frequency and Spanish-Catalan cognate status. They found a less positive-going P2 for low-frequency names compared to high-frequency names, and for names that were Spanish-Catalan cognates compared to non-cognates. Strijkers et al. (2011) make a distinction between the P2 component found in language production, which they take to be modulated by lexical properties such as word frequency, and the exogenous or visual P2 component. The authors give several reasons for assuming that the P2 found in language production tasks might reflect ease of lexical access. First, Strijkers et al. (2010) and (2011) found that the modulations of lexical frequency and cognate status on the P2 were positively correlated with reaction time data: They observed shorter naming latencies and smaller P2 components for high frequency names (compared to low frequency names) and Spanish-Catalan cognates compared to non-cognates. Second, a topographical distinction can be made between the visual P2 and the P2 modulated by lexical variables: The visual P2 and the P2 found in language comprehension tasks are typically found at fronto-central sites, whereas the lexical P2 is largest over occipital electrodes. Third, the P2 time-window (Strijkers et al., 2010: 160–240 ms; Strijkers et al., 2011: 140–210 ms) is consistent with the processing stage connected with lexical access in earlier studies (e.g., Hauk and Pulvermüller, 2004; Pulvermüller et al., 2009; see Indefrey and Levelt, 2004 and Indefrey, 2011 for reviews).

Taken together, there are clear indications that the amplitude of the P2 component can be interpreted as an index of the ease of lexical access. In terms of the present study it can therefore be reasoned that if activation of an object's features impacts the ease with which the object's name is retrieved from the lexicon, this might lead to modulation of the amplitude of the P2 component. Further, one might expect that the magnitude of the P2 modulation might be contingent upon whether the activated feature is core or peripheral to the object in question.

### Objectives

In Experiment 1 we set out to confirm earlier findings (e.g., Tanaka and Presnell, 1999; Rossion and Pourtois, 2004; Bramão et al., 2010) showing that HCD objects presented in color are recognized faster than achromatic versions of the same objects. We presented colored and achromatic pictures of HCD and LCD objects in a picture-naming paradigm. If the color-diagnosticity hypothesis put forward by Tanaka and Presnell (1999) holds, color should only affect the reaction times for HCD objects, whereas LCD items should be produced equally fast when they are presented in color compared to gray-scale. This would be in line with the frame-theoretic view that color is a core component to HCD objects, while being more peripheral to LCD objects.

Secondly, to explore whether (1) the activation of a single feature within an object's frame can impact the retrieval of that object's lexical information, and (2) whether this effect can be modulated by the fact that a feature is either core or peripheral to the object in question, we conducted a second experiment using the same stimuli as in Experiment 1. We presented the achromatic stimuli in a semantic priming paradigm preceded by appropriately colored boxes as visual prime (*primed condition*), or a black-and-white checkerboard (*unprimed condition*). Following decompositional views of conceptual representation, pre-activation of a feature should facilitate lexical access, because single features should have access to the lemma node. This would predict a less pronounced P2 and shorter reaction times for primed, HCD objects than for unprimed, HCD objects. Furthermore, we would expect no (or a smaller) effect of pre-activation of the color feature on LCD objects, resulting in no (or smaller) differences in reaction times and P2 amplitude between primed and unprimed conditions. Non-decompositional models, on the other hand, would predict no positive priming effect on HCD or LCD objects, because only the conceptual node has access to the lemma node, whereas the feature nodes connected to it do not. Frame theories could predict different outcomes depending on how they model connections between lemma nodes, concepts, and conceptual components: On the one hand, it could be possible that a single feature can be pre-activated and subsequently facilitate lexical access of, in particular, HCD objects, for which color is a core feature in the frame. On the other hand, single features might only be activated together with the whole concept, making it impossible to pre-activate them in a priming paradigm. In this case, no priming effect on HCD or LCD objects would be expected.

## Experiment 1

### Methods

#### Participants

Thirty-two participants (age range: 18–33, *M* = 25, *SD* = 4; 23 females and 9 males) took part in the experiment. All participants were right-handed native speakers of Dutch with normal or corrected-to-normal vision and no color vision impairments and received course credits or money as a reward for participation.

#### Materials

**Figure 1:**
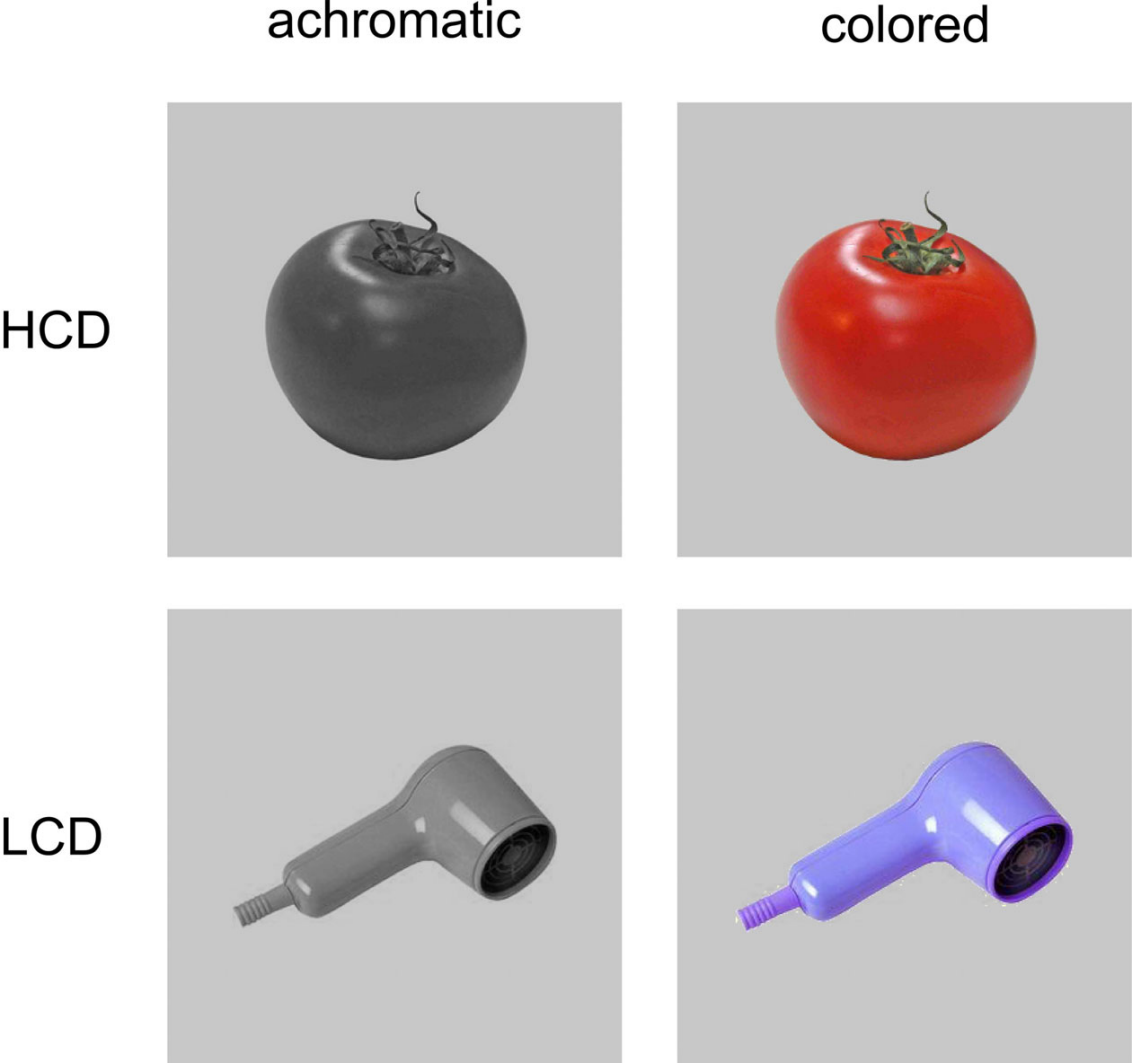
**Examples of HCD and LCD stimuli used in Experiment 1 and 2**.

One hundred and eighty colored photographs were chosen from four stimulus collections (Naor-Raz et al., 2003; Brady et al., 2008; Adlington et al., 2009; Brodeur et al., 2010). These stimulus collections were used partly because they were created for and have been previously used in studies concerned with color or color-diagnosticity (Naor-Raz et al., 2003), and because they provided high-quality photographs of the isolated objects in front of a neutral white background. All of the pictures depicted animate and inanimate objects belonging to a range of semantic categories (e.g., food, animals, vehicles, clothing, and furniture; see Figure 1 for example stimuli and Supplementary Material for a list of stimuli). Monochrome versions of all pictures were created preserving luminance values. A neutral gray background (RGB: 198, 198, 198) was added to colored and achromatic versions of the pictures.

To determine which items should be categorized as HCD and LCD, an online survey was conducted, in which 17 participants rated the appropriateness of the chosen color and the color-diagnosticity of each item by answering the following questions: “Does this object have one or more typical colors?,” “How many different colors can this object have?” and “If there is a typical color, is it the one displayed here? If not, please name the color.” On the basis of the results, the items were grouped into two sets of HCD and LCD objects. In cases where participants indicated that an item could have “one or more typical colors,” but did not agree on a common typical color in the comment field (i.e., multiple different colors were named), the item was classified as LCD. HCD and LCD items were matched for frequency and word length across sets by using lemma frequencies obtained from celex (celex database, Baayen et al., 1993; mean frequencies: HCD: *M* = 20.15 per million, LCD: *M* = 21.77 per million). HCD and LCD items were also matched for luminance (as indicated by mean gray values; HCD: *M* = 172, LCD: *M* = 173), visual complexity (comparing mean file size after compression; HCD: *M* = 18.84; LCD: *M* = 17.33) and name agreement (HCD: 92.57%, LCD: 93.74%).

Each item was shown only once to each participant. The two groups of HCD and LCD items were split in two, so that each item was presented in color to 16 participants (colored presentation mode), whereas the other 16 participants saw the achromatic version (achromatic presentation mode). We combined these items to two lists. All items shown as colored versions in list 1 (presented to 16 participants) were shown as achromatic versions in list 2 (presented to 16 other participants). The two lists were each split into four parts, which were then pseudo-randomized using the Shuffle software (Pallier, 2002) and recombined to yield the final experimental lists with equal distribution of the items throughout the experiment across subjects. Trials in which participants made no response, hesitated before giving a response (i.e., used discourse markers such as “ehm” prior to responding or prolonged the first phoneme or syllable), responded late (i.e., showed reaction times longer than the mean plus three standard deviations), did not recognize the picture (i.e., failed to use a name that can be used to describe the object), or used a possible name for the object that was not part of our balanced set of names, were excluded from the analysis^2^;.

#### Procedure

Participants were asked to name each picture as fast and accurate as possible in Dutch and to speak clearly. Stimulus presentation was controlled using the Presentation Software (Neurobehavioral Systems Inc., Albany, CA, www.neurobs.com). All pictures were presented measuring 242 by 242 px. The participants' verbal responses were recorded as wav files. Voice onset times were determined offline using Praat (Boersma, 2001) by measuring the time-window between stimulus onset (marked by a simultaneously recorded signal inaudible to the participant) and voice onset. After having completed 10 training trials, participants were given the opportunity to pose questions to the experimenter and to re-read the instructions. All experimental trials were then presented in a single block. Each training and experimental trial was of the following structure: First, a fixation cross was shown for 2000 ms, followed by a blank screen (random interval between 0 and 200 ms). The target picture was presented for 2000 ms. Between trials, a blank screen was shown for 3000 ms. The whole experiment lasted approximately 20 min.

#### Analyses

Reaction times and error rates were analyzed using a repeated-measures Analysis of Variance (ANOVA) with color-diagnosticity (HCD and LCD) and presentation mode (achromatic and colored) as within-subject factors, and using a significance criterion of *p* < 0.05.

### Results

#### Reaction times and error rates

**Figure 2:**
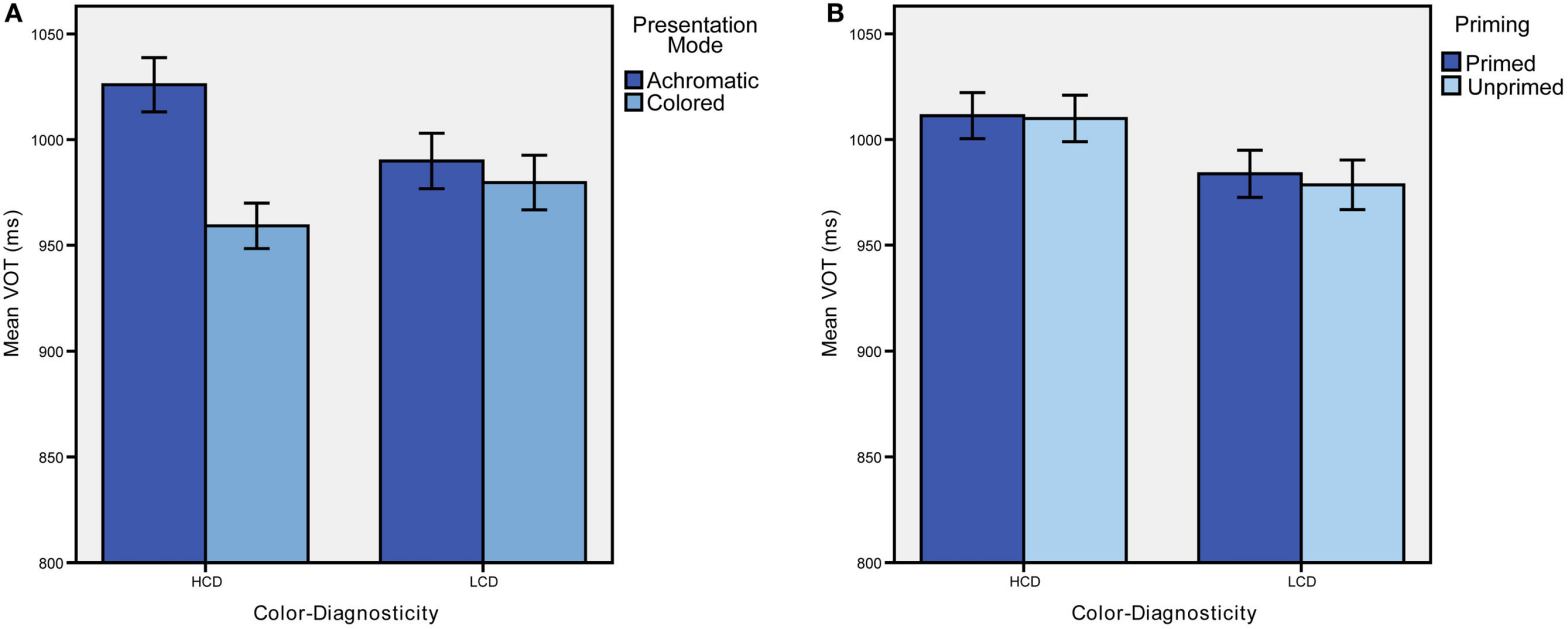
**Mean response latencies in ms obtained in Experiment 1 (A) and Experiment 2 (B)**. Error-bars represent ±1 SD.

We found a significant main effect of presentation mode [*F*_(1, 31)_ = 18.764, *p* < 0.001, η^2^ = 0.377; see Figure 2A]. There was also a significant color-diagnosticity by presentation mode interaction (see Figure 2; *F*_(1, 31)_ = 9.232, *p* = 0.005, η^2^ = 0.229). Naming latencies for HCD items were longer (*M* = 1041.308, *SD* = 201.438) than for LCD items (*M* = 994.995, *SD* = 184.538) when they were presented in gray-scale, but only a smaller difference was found between HCD and LCD objects when they were presented in color (HCD: *M* = 967.464, *SD* = 160.026, LCD: *M* = 986.449, *SD* = 200.330). Further, HCD items were recognized faster in color than in gray-scale. There was no difference for LCD items between colored and achromatic presentation mode. Trials classified as errors according to the criteria described above were excluded from the reaction time analysis (19.5% of all responses). An ANOVA with error rate as dependent variable showed no significant main effects or interactions (*p* > 0.05).

### Discussion

Our results in Experiment 1 confirm earlier findings by Tanaka and Presnell (1999) and others with respect to the effect of color on the naming of HCD and LCD objects: Whereas reaction times between HCD and LCD objects did not differ as a function of color-diagnosticity for items presented in color, naming latencies were significantly longer for HCD items when the achromatic version of the item was presented compared to colored presentation. There was no difference between colored and achromatic presentations for LCD items. These results are in line with earlier findings that suggest that color information plays an important role in recognizing and subsequently naming HCD objects.

As with previous studies concerned with color-diagnosticity, it is difficult to differentiate between early effects of color on visual processing and conceptual retrieval and later effects on lexical retrieval based on the results of Experiment 1. Therefore, in Experiment 2, we chose an experimental design that allowed us to tease apart visual processing of the color feature and lexical processing of the target's name. In order to achieve this, we made use of a semantic priming paradigm with color boxes as primes. By using a color box as a prime, processing of the (visual) color feature can be disentangled from later processing stages.

## Experiment 2

### Methods

#### Participants

A total number of 36 right-handed participants (age range: 18–62, *M* = 25, *SD* = 15, 22 females and 14 males) took part in the experiment. As in Experiment 1, all participants were native speakers of Dutch with normal or corrected-to-normal vision and no color vision impairments. After having completed the experiment, participants received course credits or money as a reward. Four participants were excluded from further analyses due to high error rates (1 participant) or because they had erroneously received the wrong experimental lists (3 participants), leaving a total of 32 participants.

#### Materials

We used the same set of gray-scale HCD and LCD pictures as in Experiment 1 (see Figure 1 for example stimuli). Matching color boxes (e.g., a yellow color box for banana) of the same size as the pictures were created using the most representative color in the picture. For LCD items, some colors were changed to other possible colors to accomplish a more balanced distribution of hues across conditions. A picture of the same size showing a black and white checkerboard pattern was created for use in the unprimed conditions.

#### Procedure

Prior to the experiment, participants were asked to fill out and sign consent and screening forms, ensuring that all requirements for participation in an EEG study were fulfilled. After electrode application, participants were tested individually in a dimly lit, acoustically shielded cabin. Instructions for the experiment were presented both in printed form and on screen prior to the experiment. In other respects, the procedure employed in Experiment 2 corresponded largely to the procedure used in Experiment 1. The same balanced experimental lists as in Experiment 1 were used. Each trial thus had the following structure: A fixation cross appeared on the screen and was presented for 2000 ms, followed by a blank screen (random interval between 0 and 200 ms). After that, the color box (in primed conditions) or checkerboard pattern (in unprimed conditions) appeared for 200 ms in the center of the screen with an ISI of 200 ms, resulting in a SOA of 400 ms. The target picture was then presented for 2000 ms. Between trials, a blank screen was shown for 3000 ms.

#### EEG recording

**Figure 3:**
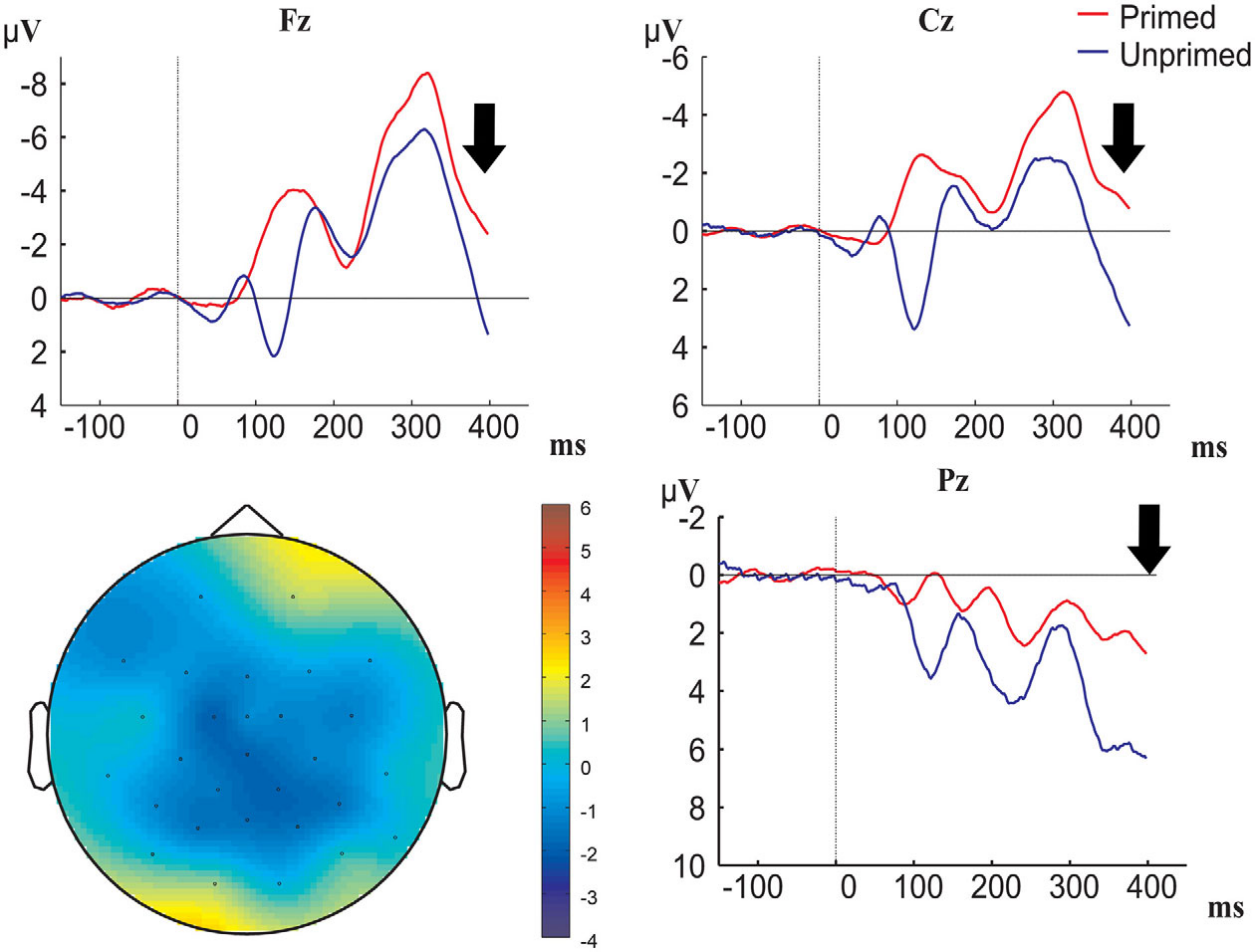
**Grand-averaged ERP waveforms time-locked to prime onset (primed conditions are represented in red, unprimed conditions in blue; baseline between −150 and 0 ms before prime onset) at electrodes Fz, Cz, and O1, and topographical map of prime effect**. Black arrow denotes target onset.

The EEG was continuously recorded from 32 tin electrodes using an active electrode cap (ActiCap). 27 scalp electrodes were embedded in the cap at the following sites: Fp1, Fp2, F7, F3, Fz, F4, F8, FC5, FC1, FCz, FC2, FC6, T7, C3, Cz, C4, T8, CP5, CP1, CP2, CP6, P7, P3, Pz, P4, P8, O1, O2 (see Figure 3 for electrode positions on the scalp). Three additional electrodes were placed on the participant's face to register vertical and horizontal EOGs. Scalp electrodes were referenced on-line to the left mastoid (REF), and re-referenced offline to linked mastoids. Prior to recording, impedances were reduced to 20 kΩ or less with the amplifier set up for high-impedance measurement (amplifier impedance 10 MΩ; cf., Ferree et al., 2001). The EEG and EOG recordings were amplified with a BrainAmp DC amplifier (Brain Products, München, Germany). The recordings were sampled at 500 Hz with a high cut-off filter at 125 Hz and a low cut-off filter with a time constant of 10 s. Trials with eye-blinks or deflections exceeding 100 μ V were rejected. In total, 33% of experimental trials were rejected in this manner. The ERP data were analyzed after artifact rejection, averaging and baseline correction.

#### Behavioral analyses

Voice onset times and errors rates were determined using the same procedures as in Experiment 1. We analyzed reaction times and error rates using a repeated-measures ANOVA with color-diagnosticity (HCD and LCD) and priming (primed and unprimed) as within-subject factors with a significance criterion of *p* < 0.05.

#### ERP analyses

Because visual properties of the prime could potentially influence the baseline period of the target, we first checked whether the visual properties of the prime (checkerboard or color prime) led to differences in the ERP waveforms. And, more importantly, whether these differences carried over to the baseline interval of the target. We used a baseline in the −150 to 0 ms interval before prime onset and compared waveforms between primed and unprimed conditions.

Repeated-measures ANOVAs were carried out over the mean ERP amplitudes from the grand average, target-locked, ERP data in time-windows centered on the components of interest. The ANOVAs included the factors: color-diagnosticity (HCD and LCD), priming (primed and unprimed), anteriority (frontal, midline and posterior), and laterality (left, central and right). Again, a significance criterion of *p* < 0.05 was used. Significance levels were reported after Greenhouse–Geisser correction (Greenhouse and Geisser, 1959). The time-windows were chosen after visual inspection of the data and based on previous literature by Strijkers et al. (2010). Electrode locations were recoded into two factors according to their *anteriority*, that is, either: frontal (Fp1, Fp2, F7, F3, Fz, F4, F8, FC5, FC1, FCz, FC2, FC6), midline (T7, C3, Cz, C4, T8), or posterior (CP5, CP1, CP2, CP6, P7, P3, Pz, P4, P8, O1, O2); and their *laterality*, that is, either: left (Fp1, F7, F3, FC5, FC1, T7, C3, CP5, CP1, P7, P3, O1), central (Fz, FCz, Cz, Pz), or right (Fp2, F4, F8, FC2, FC6, C4, T8, CP2, CP6, P4, P8, O2). Trials classified as errors were excluded from the analyses.

### Results

#### Behavioral results

Reaction times differed significantly between HCD and LCD conditions [*F*_(1, 31)_ = 6.414, *p* = 0.017, η^2^ = 0.171; see Table 1 for mean reaction times and error rates]. Notably, naming latencies for HCD items were significantly longer than for LCD items (see Figure 2B). Priming, however, did not yield any significant effects.

**Table 1:** **Mean response latencies in ms and error rates, SD in parentheses**.

	**HCD items**		**LCD items**	
	**RT**	**Error rates**	**RT**	**Error rates**
Primed	1013.2 (136.1)	0.20 (0.08)	983.9 (149.2)	0.16 (0.09)
Unprimed	1011.8 (150.7)	0.19 (0.09)	979.9 (157.8)	0.17 (0.08)

Erroneous trials were defined according to the criteria mentioned above (17.9% of all responses). Similar to the pattern found in the reaction time analysis, error rates were significantly higher for HCD items compared to LCD items, [*F*_(1, 31)_ = 5.486, *p* = 0.026]. There were no other main effects or interactions (*p* > 0.05).

#### ERP results

**Figure 4:**
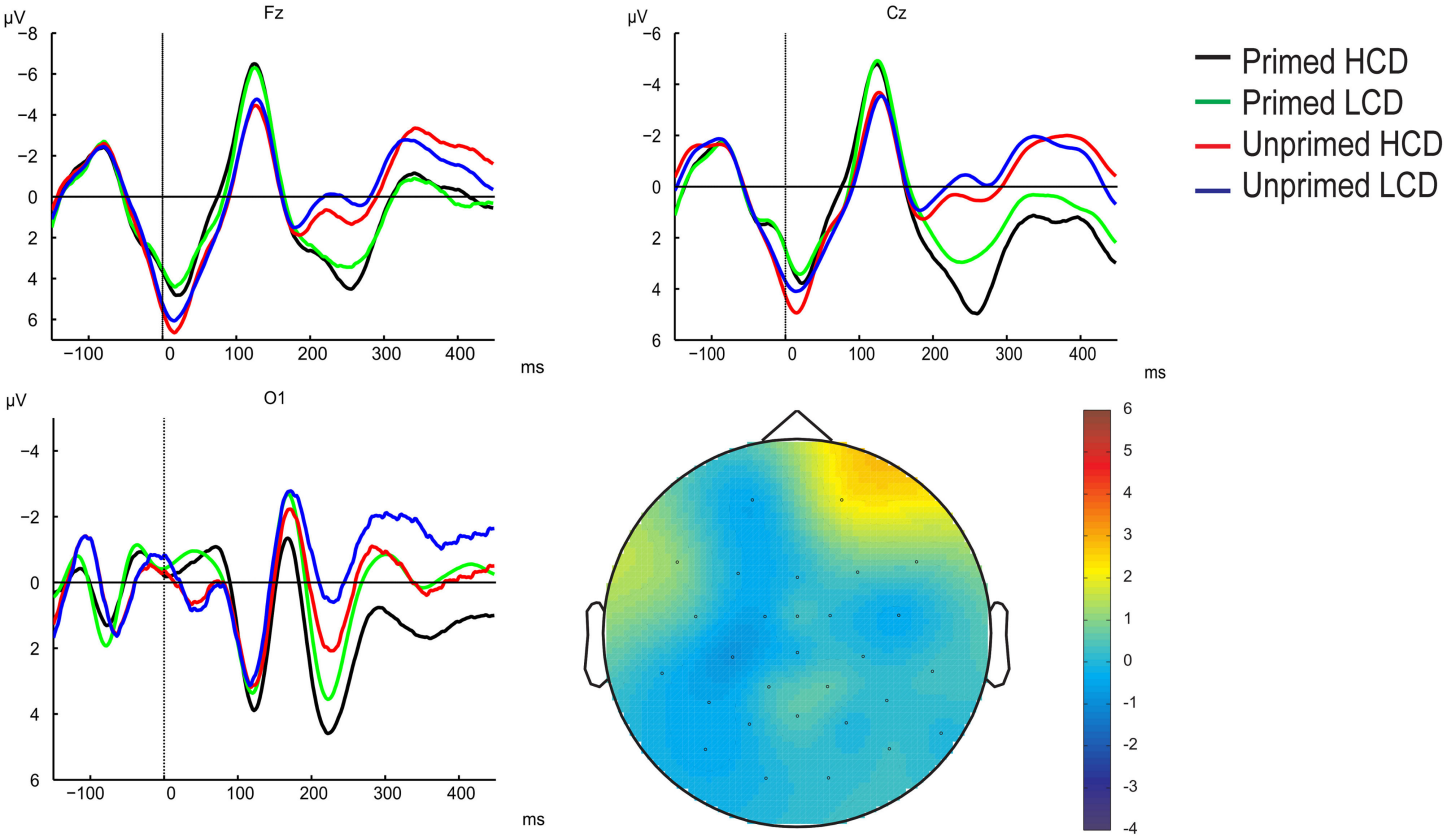
**Grand-averaged ERP waveforms time-locked to target onset (baseline between −150 and 0 before target onset) for primed HCD (black), primed LCD (green), unprimed HCD (red), and unprimed LCD (blue) conditions at electrodes Fz, Cz, and O1, and topographical map of the 0–100 ms time window showing the prime effect**.

**Figure 5:**
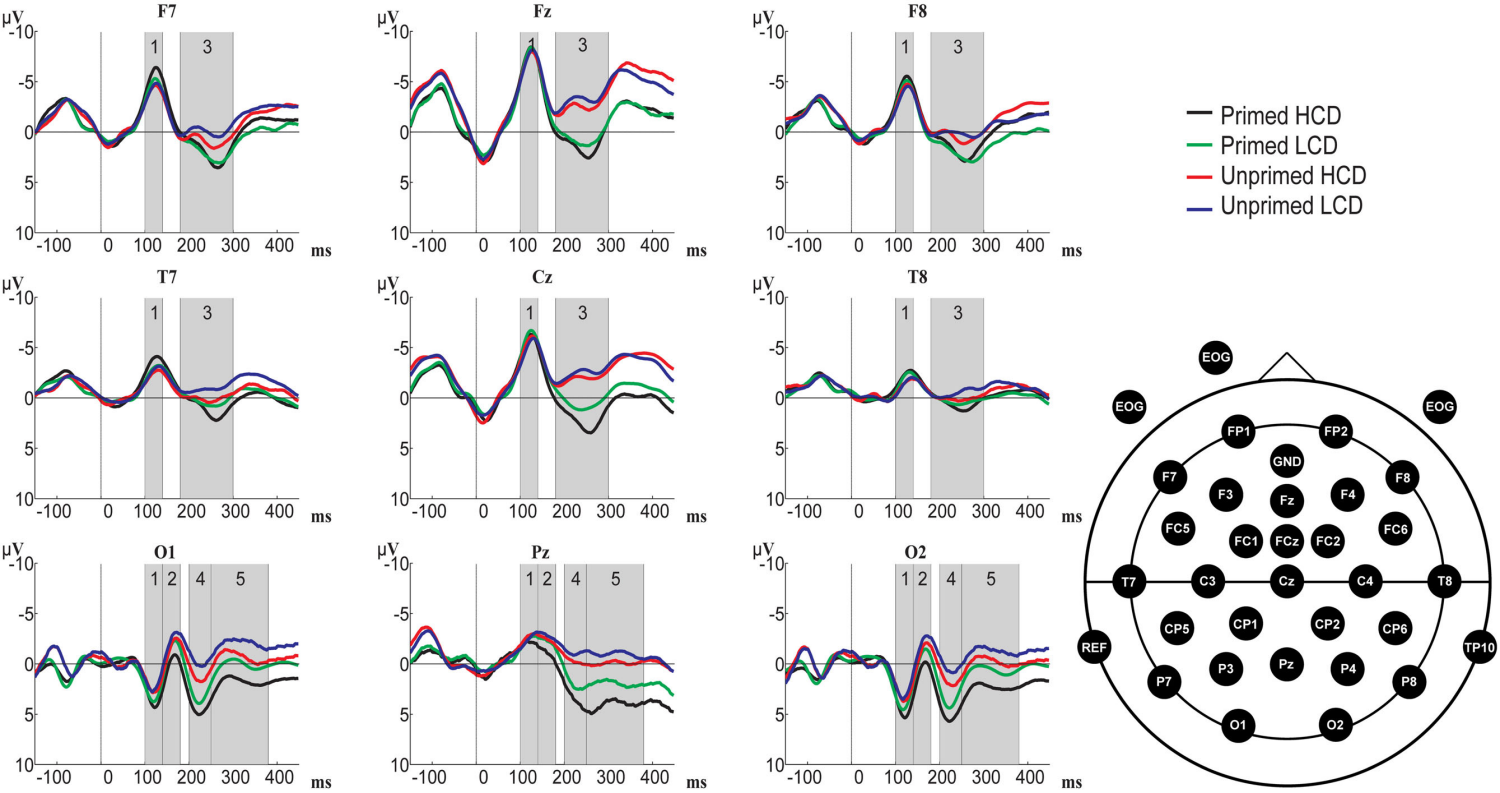
**Overview of grand-averaged ERP waveforms (baseline corrected using the 0–100 ms interval) for primed HCD (black), primed LCD (green), unprimed HCD (red), and unprimed LCD (blue) conditions at example electrodes across the scalp and electrode positions**. Numbered gray areas indicate time-windows chosen for analysis (1:100–140 ms, 2:140–180 ms, 3:180–300 ms, 4:200–250 ms, 5:250–380 ms).

In Figure 3 we see that processing of the prime indeed had an effect, namely the waveforms for checkerboards (i.e., the unprimed condition) were negatively shifted compared to the waveforms for color boxes (i.e., the primed condition). Figure 4 shows that this negative polarity shift for primed vs. unprimed conditions still exists at target onset, and potentially continues into the P1-N1 latency range. In order to control for the spurious priming effects that are caused by carry over from the prime we applied a new baseline correction in the 0 to 100 ms interval after target onset. In Figure 5, we can see that this baselining procedure corrected for the effect of priming in the P1 latency range (which was likely then due to carry-over) on frontal and central electrodes, but revealed an effect on posterior electrodes. We also see that posterior electrodes exhibit ERP components which are not visible on frontal and central sites. On posterior electrodes there is a positive-going component between 100 and 140 ms followed by a negative going component in the time-window between 140 and 190 ms, further we see a positive-going component between 200 and 250 ms followed by a negative component from 250 to 380 ms. On frontal and central sites there is only a negative going component between 100 and 140 ms followed by a positive going component between 180 and 300 ms. To test whether any of the components exhibited significant differences between conditions we performed a repeated-measurements ANOVA with priming (primed or unprimed), color-diagnosticity (HCD or LCD), anteriority (anterior, midline, or posterior), and laterality (left, midline, or right) as factors on all electrode sites in the time-window from 100 to 140 and from 180 to 300 ms, and additional ANOVAs with priming, color-diagnosticity and laterality as factors in the time-window from 100 to 140 ms, 140 to 180 ms, 200 to 250 ms, and 250 to 380 ms on posterior electrode sites, and from 100 to 140 ms and 180 to 300 ms on frontal electrode sites and the midline.

***Whole scalp analysis***. 100–140 ms: There were no significant main effects in this time-window (*p* > 0.05; for grand-averaged waveforms see Figure 5). We found an interaction between priming and anteriority, showing that at frontal and central electrodes, primed items elicited a larger negativity than unprimed items, whereas on posterior sites, priming resulted in more a positive waveform [*F*_(2, 62)_ = 6.638, pGG = 0.002, η^2^ = 0.176]. Further, we observed a color-diagnosticity by priming by laterality interaction [*F*_(2, 62)_= 3.727, *p* = 0.030, η^2^ = 0.107]. On left electrode sites, HCD items (primed: *M* = −3.631, *SD* = 3.645; unprimed: *M* = −3.037, *SD* = 3.884) were more affected by priming than LCD items (primed: *M* = −3.202, *SD* = 3.830, unprimed: *M* = −3.066, *SD* = 3.554).

**Figure 6:**
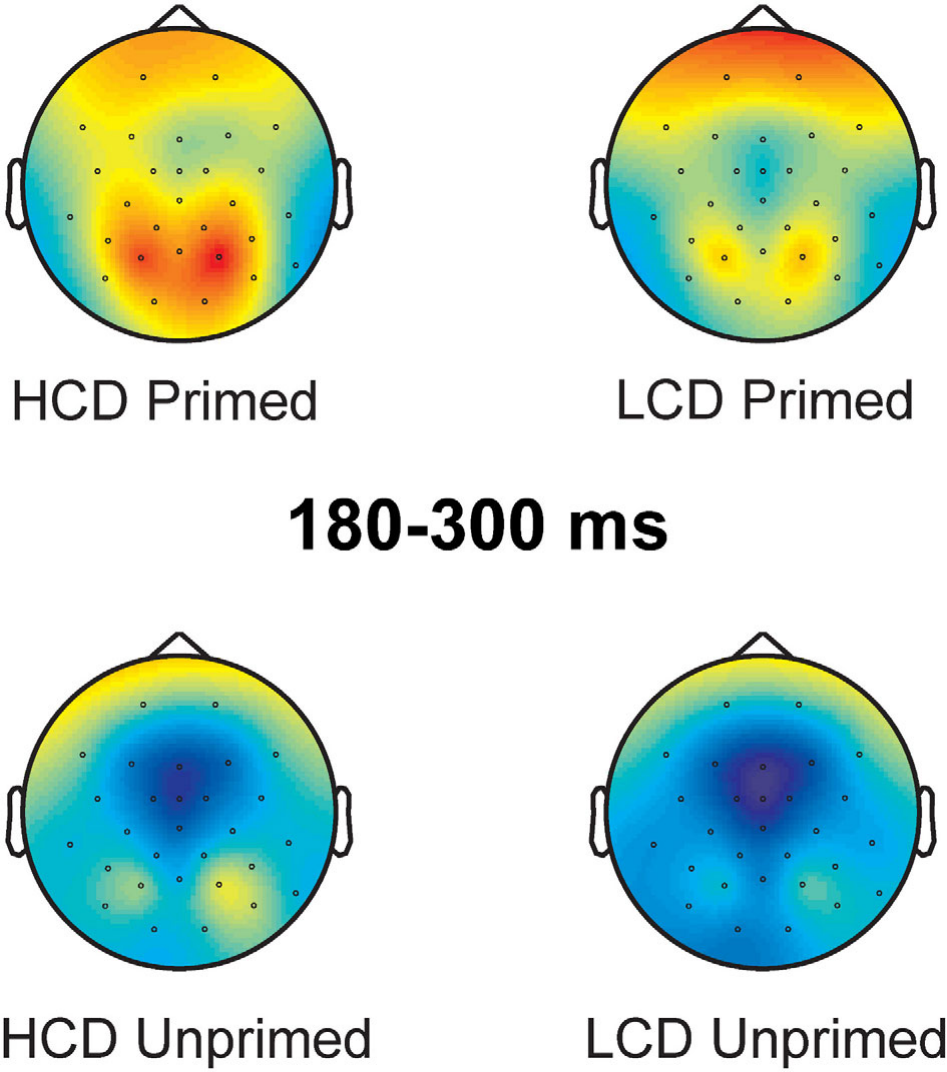
**Scalp topography of the 180–300 ms time-window (baseline between 0 and 100 ms post-target onset)**.

180–300 ms: In this time-window, the ANOVA yielded main effects of color-diagnosticity [*F*_(1, 31)_ = 12.734, *p* = 0.001, η^2^ = 0.291] and priming [*F*_(1, 31)_ = 41.327, *p* < 0.001, η^2^ = 0.571; for scalp topographies see Figure 6], HCD items and primed items eliciting more positive waveforms than LCD and unprimed items. The largest difference between HCD and LCD conditions emerged at posterior sites, as revealed by an interaction between color-diagnosticity and anteriority [*F*_(2, 62)_ = 9.085, *pGG* = 0.004, η^2^ = 0.227]. Further, we found an interaction between priming and laterality [*F*_(2, 62)_ = 19.871, *pGG* < 0.001, η^2^ = 0.391], as well as a three-way interaction between priming, laterality and anteriority [*F*_(4, 124)_ = 7.219, *pGG* < 0.001, η^2^ = 0.189]. At both left and right electrodes, the difference between primed and unprimed was smaller at the midline than at frontal and posterior sites. We also observed interactions between color-diagnosticity, priming and anteriority [*F*_(2, 62)_ = 2.838, *pGG* = 0.045, η^2^ = 0.084], and between color-diagnosticity, priming, and laterality [*F*_(2, 62)_ = 4.361, *p* = 0.017, η^2^ = 0.123], showing that priming had a stronger influence on HCD items compared to LCD items particularly on posterior (primed HCD: *M* = 3.225, *SD* = 2.939; unprimed HCD: *M* = 0.287, *SD* = 3.775; primed LCD: *M* = 1.611, *SD* = 3.193; unprimed LCD: *M* = −0.622, *SD* = 4.079) and central sites (primed HCD: *M* = 2.144, *SD* = 4390; unprimed HCD: *M* = −1.694, *SD* = 4.921; primed LCD: *M* = 0.791, *SD* = 4.211; unprimed LCD: *M* = −2.335, *SD* = 5.007).

***Posterior electrode sites***. 100–140 ms: No significant main effects were present in this time-window. We observed an interaction between color-diagnosticity, priming, and laterality [*F*_(2, 62)_ = 3.343, *p* = 0.042, η^2^ = 0.097]. At central electrodes, waveforms elicited by primed, HCD items (*M* = −2.058, *SD* = 3.176) were less negative than those elicited by unprimed, HCD items (*M* = −2.664, *SD* = 3.418), whereas there was a smaller difference between primed and unprimed LCD objects (primed: *M* = −2.368, *SD* = 3.319; unprimed: *M* = −2.414, *SD* = 2.829).

140–190 ms: In this time-window, we observed main effects of color-diagnosticity [*F*_(1, 31)_ = 12.065, *p* = 0.002, η^2^ = 0.280] and priming [*F*_(1, 31)_ = 8.043, *p* = 0.008, η^2^ = 0.206]. LCD and unprimed items resulted in more negative waveforms compared to HCD and primed items.

200–250 ms: Significant main effects were present for color-diagnosticity [*F*_(1, 31)_ = 19.834, *p* < 0.001, η^2^ = 0.390] and priming [*F*_(1, 31)_ = 33.994, *p* < 0.001, η^2^ = 0.523]. HCD items and primed items elicited more positive waveforms than LCD and unprimed items. We also observed an interaction between priming and laterality [*F*_(2, 62)_ = 8.174, *p* = 0.001, η^2^ = 0.209], showing that the priming effect was stronger on central and left electrode sites compared to right sites. There was a trend toward an interaction between color-diagnosticity, priming, and laterality [*F*_(2, 62)_ = 3.379, *pGG* = 0.052, η^2^ = 0.098], indicating that HCD items were particularly affected by priming compared to LCD items at central electrodes (primed HCD: *M* = 3.360, *SD* = 3.659; unprimed HCD: *M* = −0.346, *SD* = 4.824; primed LCD: *M* = 1.912, *SD* = 3.827; unprimed LCD: *M* = −1.164, *SD* = 5.153).

250–380 ms: In this time-window, the ANOVA yielded main effects of color-diagnosticity [*F*_(1, 31)_ = 33.367, *p* < 0.001, η^2^ = 0.518] and priming [*F*_(1, 31)_ = 34.750, *p* < 0.001, η^2^ = 0.529]. HCD and primed items resulted in more positive waveforms compared to LCD and unprimed items. Further, there was an interaction between color-diagnosticity, priming, and laterality [*F*_(2, 62)_ = 3.846, *p* = 0.027, η^2^ = 0.110]. Particularly at central electrodes, there was a larger difference between primed, HCD (*M* = 1.304, *SD* = 5.521) and unprimed, HCD conditions (*M* = −2.800, *SD* = 5.692) than there was between primed (*M* = −0.085, *SD* = 5.084) and unprimed (*M* = −3.210, *SD* = 5.589) LCD conditions.

***Frontal and midline electrode sites***. 100–140 ms: There were no significant main effects in this time-window (*p* > 0.05). Color-diagnosticity interacted with priming and laterality [*F*_(2, 62)_ = 3.483, *p* = 0.037, η^2^ = 0.101]. At left electrode sites, HCD items (primed: *M* = −5.247, *SD* = 2.806; unprimed: *M* = −4.277, *SD* = 3.735) were particularly affected by priming compared to LCD items (primed: *M* = −4.654, *SD* = 3.289; unprimed: *M* = −4.318, *SD* = 3.385), primed items resulting in a larger negativity than unprimed items.

180–300 ms: We found significant main effects of color-diagnosticity [*F*_(1, 31)_ = 5.761, *p* = 0.023, η^2^ = 0.157] and priming [*F*_(1, 31)_ = 38.915, *p* < 0.001, η^2^ = 0.557]. HCD conditions elicited more positive waveforms compared to LCD conditions. We also observed an interaction between priming and laterality [*F*_(2, 62)_ = 21.245, *p* < 0.001, η^2^ = 0.407], the difference between primed and unprimed items being most pronounced at central electrodes. Further, there was an interaction between color-diagnosticity, priming and laterality [*F*_(2, 62)_ = 3.692, *p* = 0.031, η^2^ = 0.106], showing that at central electrodes, priming had a stronger effect on HCD items (primed: *M* = 1.562, *SD* = 4.749; unprimed: *M* = −2.341, *SD* = 5.085) than it had on LCD items (primed: *M* = 0.376, *SD* = 4.454; unprimed: *M* = −2.870, *SD* = 5.081).

### Discussion

In Experiment 2, we found a behavioral effect of color-diagnosticity (HCD items were produced on average 31 ms slower than LCD items), but no effect of priming. The ERPs revealed an early interaction between color-diagnosticity and priming in the 100–140 ms time-window, where ERPs elicited for primed HCD items elicited the most pronounced P1. In the three following time-windows, both priming and HCD resulted in a more positive waveform. We observed further interactions between color-diagnosticity and priming on the P2 at fronto-central electrodes (180–300 ms), and a trend for an interaction in the same direction at posterior electrodes (200–250 ms). The P2, which has been shown to be an index of difficulty of lexical retrieval by, e.g., Strijkers et al. (2010), was most positive for primed, HCD items. Although the behavioral results yielded no effect of priming, the priming effect on the P2 could be taken to suggest that lexical retrieval is hindered by color priming. We will discuss this issue in more detail below. Color-diagnosticity and priming also interacted in the latest time-window analyzed at posterior electrodes (250–380 ms). Again, HCD, primed items resulted in more positive waveforms. Possible accounts for these findings and theoretical implications will be discussed below.

## General discussion

Our behavioral results in Experiment 1 showed that achromatic versions of HCD objects were named more slowly than colored versions of HCD objects, whereas no such difference was found between colored and achromatic presentations of LCD objects. In Experiment 2, HCD resulted not only in slower object naming, but also a more positive P2 component. Priming also elicited a more pronounced P2 component, but had no effect on naming latencies. Lastly, the effect of priming was stronger for HCD objects than for LCD objects, as revealed by an interaction between priming and color-diagnosticity in early (P1) and later processing stages (P2 and N300). The interaction between color-diagnosticity and priming on the P1 held irrespective of whether electrodes on the whole scalp were considered or only electrodes belonging to either frontal and central or posterior sites. The interaction effect on the P2 was most evident in the whole scalp analysis in the 180–300 ms time-window and on frontal and central electrode sites. The effect also approached significance when we analyzed the isolated P2 peak on posterior sites. In all three time-windows, waveforms for HCD items were more pronounced for primed compared to unprimed conditions, whereas smaller differences between primed and unprimed LCD items were found.

### Early effects (P1 and N1)

The P1-N1 complex has been associated with visual processing (influenced by stimulus characteristics such as visual complexity and luminance; Johannes et al., 1995), and can be modulated by spatial attention (e.g., Mangun et al., 1993; Luck et al., 2000). In the present study, we found a more positive deflection for primed, HCD conditions on the P1-N1 complex, resulting in an interaction between color-diagnosticity and priming on the P1 and main effects of priming and color-diagnosticity on the N1. Since there were no statistically significant differences between HCD and LCD stimuli in visual complexity and luminance, it is unlikely that these early effects reflect differences in early visual processing between HCD and LCD objects. A possible account for the main effect of color-diagnosticity could be that the visual recognition of HCD objects depends more on color. HCD objects tend to represent natural objects, which often exhibit higher intra-categorical structural similarity (Tanaka and Presnell, 1999; Tanaka et al., 2001; Laws and Hunter, 2006) than artificial objects do (consider, e.g., tomatoes and oranges, which are both round objects). For HCD items an important (and often distinctive) visual feature is missing (color) from our achromatic stimuli. This likely rendered these objects more difficult to recognize compared to LCD objects. This account would also fit with our behavioral data from Experiment 1, which show slower RTs to achromatically presented HCD objects compared to colored HCD objects. The interaction on the P1 suggests that priming has a different effect on high compared to LCD items, because primed, HCD items resulted in the most positive waveforms. At this point, however, it is unclear whether the nature of this effect is facilitatory or inhibitory. We will come back to this question when discussing the interactions found in the P2 and N300 latency range.

### P2

The P2 component has been shown by Strijkers et al. (2010) and others (Costa et al., 2009; Strijkers et al., 2011, 2013) to be modulated by lexical retrieval in overt language production. More specifically, lexical items that are harder to access (e.g., low frequency words) elicit a larger posterior P2 component than lexical items that allow for easier access (e.g., high frequency words). The slower reaction times found in Experiments 1 and 2 and the more pronounced P2 component for HCD items obtained in Experiment 2 suggest that the names of HCD objects are more difficult to retrieve. Thus, high color-diagnosticity modulates the P2 component, and does not seem to help lexical access at this point. Instead, our results indicate that high color-diagnosticity hinders lexical access. Previous studies involving HCD and LCD stimuli found that HCD objects (which tend to belong to natural categories such as fruits or vegetables) are harder to recognize than LCD objects (which tend to be man-made) when presented as achromatic pictures (e.g., Tanaka and Presnell, 1999). This fits well with our behavioral data obtained in Experiment 1, which also showed that the reaction times for HCD objects were more affected by achromatic presentation than the reaction times for LCD objects. This fact yields a possible avenue for interpreting the main (detrimental) effect of color-diagnosticity: For HCD objects (more natural), many similar items are activated alongside the intended item at the conceptual stage, e.g., a picture of an orange might also activate the concept of apple which is visually similar. This is less of an issue for LCD (more artificial) items where there are fewer visual similarities (e.g., between bicycles and umbrellas). This difference might have consequences for lexical access: More competing lemmas are activated for HCD objects, making selection of the right lemma more difficult. For LCD objects, there are less competitors, or their competitors are not as strongly activated (because the objects do not share as many visual features). Therefore, activation levels of competitors for lexical access are not increased as much for LCD items. Thus, similar to previous studies (e.g., Costa et al., 2009; Strijkers et al., 2010, 2011, 2013) the P2 effect for color-diagnosticity seems to be indicative of a more effortful lexical retrieval for high compared to LCD items.

We also found a main effect of priming in the same direction as the main effect of color-diagnosticity, with more positive waveforms for primed items. This effect might be interpreted in a similar way, if the P2 is taken as an index of lexical retrieval. Following this interpretation, priming with a color box would also be detrimental for lexical access. A possible account for more difficult lexical retrieval of primed items is that the color boxes used as a prime activate larger cohorts of words that share this color. Thus, a red box activates the adjective *red*, but also nouns like *tomato*, *fire truck*, or *lipstick*, which are competitors to the target word. A checkerboard, on the other hand, likely activates far less associatively or semantically related nouns (such as, for instance, *chess*, *game*, or *queen*). It is also possible that color priming can be detrimental to picture naming if the color chosen as visual prime does not fully correspond to the individual participant's specific representation of the color in the object's frame. The color shown to pre-activate the target strawberry, for instance, might correspond more closely to the value of the color feature present in the individual participant's frame for tomato, thus activating the concept tomato (and other objects represented with this value of the color feature). This increase of competition at the conceptual level would consequently influence lexical retrieval at a later stage as reflected in a more pronounced P2 for primed items. This interpretation is, of course, contingent upon the magnitude of the P2 being an index of the effort involved in the lexical retrieval process, which is well supported by the currently available literature (e.g., Costa et al., 2009; Strijkers et al., 2010, 2011, 2013). Importantly, we also have to take into account the fact that our behavioral data showed no significant effect of priming.

A possible reason for the absence of a behavioral priming effect is that the naming latencies we observed were quite long (on average 997 ms, probably due to lack of repetition of the items and a familiarization procedure prior to the experiment). Subtle differences between primed and unprimed conditions may, thus, be lost due to a ceiling effect and might only be visible in the ERPs^3^. However, given that the effects of color-diagnosticity and priming on the P2 are of a similar magnitude it is unclear why the color-diagnosticity effect would have behavioral consequences while the priming effect does not. This makes a second possibility more plausible, namely that the detrimental effect of priming during lexical access (i.e., on the P2) is compensated by a facilitatory effect of priming during object recognition (i.e., on the P1-N1 complex). This facilitatory effect might arise due to the additional color information provided by the color prime being helpful for the visual recognition of HCD objects.

Following the interpretation of the main effects of priming and color-diagnosticity outlined above, it seems plausible that the interaction between color-diagnosticity and priming on the P2 indicates that priming has an influence on lexical access of, in particular, HCD objects. This could be explained by increased lexical competition: priming with a color box creates lexical competition by activating concepts associated with that particular color. This competition is largest in the case of HCD items, where the competition between similar concepts activated by a particular color prime might be stronger than for LCD items (e.g., a red box might activate multiple kinds of red, HCD items, i.e., red fruit such as tomato, strawberry, raspberry, or cherry). Alternatively, if we interpret the main effect of priming as a result of a discrepancy between the color prime and the mental representation of the color feature, this could also account for the interaction between color-diagnosticity and priming. The difference between the color of the prime and the represented color might place additional processing demands on lexical retrieval of HCD objects, for which color is an important visual cue. Consequently, the lexical representations of HCD objects would be more difficult to retrieve compared to those of LCD objects. The present data, however, cannot distinguish between these lines of explanation.

To summarize, the P2 component has been shown to reflect lexical retrieval (Costa et al., 2009; Strijkers et al., 2010, 2011). Therefore, we suggest that the more pronounced P2 component for HCD and for primed items (compared to LCD and unprimed items) found in Experiment 2 might reflect the fact that these items require a more effortful lexical retrieval. Particularly HCD objects were affected by priming in this time-window, which we can explain by either increased lexical competition or the consequences of a discrepancy between the color used as a prime and the individual participant's representation of the color feature of the object.

### N300

Previous research has connected the N300 component to perceptual as well as semantic processing (e.g., Barrett and Rugg, 1990; McPherson and Holcomb, 1999). In a verification task involving HCD and LCD objects, in which participants had to indicate whether a written color name (e.g., “*red*”) matched the typical color of the target picture (e.g., “strawberry”), Bramão et al. (2011a) found an effect of color knowledge in this time-window (N350). According to Bramão et al. (2011a), their results suggest that this time-window is crucial for the integration of shape and color information, which can then be used to access structural representations of the object in long-term memory. Bramão et al. (2011a) also point to previous literature showing that the N350 is “related to the selection of a stored structural description model to match against the perceptual input” (p. 35; citing Schendan and Kutas, 2003). Bramão et al., (2012) suggest that the additional color information might be used to narrow down the number of possible structural features used to identify the object.

Additional support for the view that the N300 component might be related to semantic integration of visual features comes from Lu et al. (2010). Their stimuli consisted of congruently colored, incongruently colored and achromatic pictures of HCD objects. The experimental task required the participants to detect repetitions of a number of filler items. In the ERPs, Lu et al. (2010) found that black-and-white and atypically colored objects were associated with more negative N300 amplitudes compared with typically colored objects. The authors conclude that at this processing stage, knowledge about typical colors of HCD objects is integrated in semantic memory. Note that Bramão et al. (2012) did not find an effect of color on the N300 in their delayed picture-naming task with colored and achromatic objects. Bramão et al. (2012) did, however, find an effect of color for HCD objects in the N400 time-window, which is also connected to the processing of semantic knowledge (e.g., Hamm et al., 2002), suggesting that color information is relevant for the semantic processing of these objects.

In the present study we obtained an interaction between color-diagnosticity and priming in the N300 time-window, reflecting the fact that color priming resulted in larger N300 amplitudes; particularly so for HCD items. This interaction might be interpreted as reflecting visual feature processing, in line with earlier studies. Based on the present data, however, we can only speculate about the exact functional nature of the N300. For HCD items (that do have a color feature specified), participants may, for instance, attempt a matching between the prime's color and the target's color attribute as an additional conceptual monitoring process (note that the outcome of such a matching might indirectly help lexical selection by strengthening the activation of the target concept). This is to be distinguished from processes operating at the lemma level as such (which we consider to be indexed by the P2).

In summary, previous research suggests that the N300 reflects the processing of visual semantic features. These findings fit well with the interaction between color-diagnosticity, priming and laterality that we found in the N300 time-window. HCD, primed items elicited the most positive-going waveform compared to our other experimental conditions, suggesting that the additional color information provided by the color prime places greater demands on visual semantic processing for, in particular, HCD items. Further studies are necessary to determine the exact locus of the N300 component within the language production process but, whether or not the N300 reflects lexical or post-lexical processing, the observed interactions between color-diagnosticity and priming on the N300 and the earlier P2 have interesting implications for how we conceive of color representations in object frames.

### Implications for theories of semantic representations

Following decompositional views of semantic representations, conceptual features (such as a particular color in the case of HCD objects) should have access to the lemma node of the corresponding concept. Thus, activation of these features would be beneficial for lexical retrieval. This account would predict a facilitatory effect of priming on HCD objects, for which color is a relevant feature. LCD objects, which do not have a typical color, should not be affected by color priming. These predictions do not fit with our results, which showed a detrimental, rather than facilitatory, effect of priming on HCD objects. Our results are compatible with a classic non-decompositional view or with a frame-theoretical view, in which an object's attributes are only accessed via its corresponding conceptual node and cannot themselves activate the object's lexical lemma. Both accounts would not predict a facilitatory effect of color primes because only concept nodes (but not conceptual features) have access to the relevant lemma. Although both accounts as such would not predict the observed inhibitory effect of color primes on HCD objects, the interaction between priming and color-diagnosticity found in the ERPs could be explained (1) by additional lexical competition or (2) by interference with the activation of HCD concepts due to a mismatch between stored and perceived color. Based on the present data, it is not possible to distinguish between these two possible explanations. Note, however, that the second explanation relies on frame-theoretical conceptual representations allowing for a specification of the particular shade of a color of a particular concept, e.g., the yellow of a lemon. Further experiments testing the second explanation might therefore adjudicate on the necessity of frame-theoretical representations.

### Conclusions

Taken together, our results show that lexical access of, in particular, HCD objects, is hindered by the presence of a color prime. This finding indicates that, contrary to decompositional accounts of semantic representations, there is no direct link between the conceptual features of an object (such as color) and its corresponding lemma node. Our findings have consequences for frame-theoretical accounts of conceptual representations, namely that only the central node of a concept should be used for lexical access. Further, the interaction between priming and color-diagnosticity in the N300 time-window might suggest the additional involvement of a monitoring process that assesses the fit of available visual semantic features.

### Conflict of interest statement

The authors declare that the research was conducted in the absence of any commercial or financial relationships that could be construed as a potential conflict of interest.

## Footnotes

^1^As a convention, we use small capital letters for concepts (cat) and italics for attributes and values in frames (*color—yellow*).

^2^On the basis of these criteria, 60 items were excluded from the analysis based on the data obtained in Experiment 2, resulting in a final set of 120 items which was analyzed in both experiment. Before and after creating the final list of 120 items, the HCD and LCD items were matched. For the analysis of Experiment 1, we also excluded items for which the achromatic version was not sufficiently different from the colored version (e.g., tooth; 7 items).

^3^A number of previous studies have also obtained ERP effects in the absence of corresponding behavioral effects (see, e.g., Osterhout et al., 2004; Qu et al., 2012).
