# Supplementary material for: The use of conceptual components in language production: an ERP study
Source: Front Psychol. 2014 Apr 29;5:363. doi: 10.3389/fpsyg.2014.00363 (PMC4010786; doi:10.3389/fpsyg.2014.00363)
Supplement: Supplementary file 1 [file DataSheet1.DOCX]

***Supplementary Material***

# The use of conceptual components in language production:

# an ERP study

**Alexandra Redmann^1,2^, Ian FitzPatrick^1,2^, Frauke Hellwig^1,2^, Peter Indefrey^1,2^**

^1^ Institut für Sprache und Information, Abteilung für Allgemeine Sprachwissenschaft, Heinrich Heine Universität Düsseldorf, Düsseldorf, Germany

^2^ Donders Institute, Centre for Cognitive Neuroimaging, Radboud University Nijmegen, Nijmegen, Netherlands

*** Correspondence:** Alexandra Redmann, Institut für Sprache und Information, Abteilung für Allgemeine Sprachwissenschaft, SFB – Projekt A04, Heinrich Heine Universität Düsseldorf, Kruppstraße 108, 40227 Düsseldorf, Germany. E-mail: aredmann@phil.uni-duesseldorf.de.

1. **Supplementary Tables**

**Supplementary Table 1.** List of high color-diagnostic items used in Experiments 1 and 2 (translations in parentheses). For each item, we present a color adjective describing the color box used as a prime together with RGB-values of the color box, as well as the color-diagnosticity rate (CD; i.e., proportion of participants that consider the item to have a typical color) obtained in the pre-study.

| **High color-diagnostic items** | | | | | | | | | | | |
| --- | --- | --- | --- | --- | --- | --- | --- | --- | --- | --- | --- |
| **Name** | **Color** | **R** | **G** | **B** | **CD** | **Name** | **Color** | **R** | **G** | **B** | **CD** |
| aardappel (potato) | brown | 227 | 193 | 145 | 1,0 | kokosnoot (coconut) | brown | 146 | 91 | 34 | 1,0 |
| aardbei (strawberry) | red | 200 | 22 | 30 | 0,9 | kreeft (lobster) | red | 215 | 6 | 0 | 1,0 |
| ananas (pineapple) | yellow | 220 | 181 | 59 | 0,9 | krokodil (crocodile) | green | 59 | 87 | 64 | 0,9 |
| aubergine (eggplant) | purple | 86 | 38 | 38 | 0,9 | kuiken (chick) | yellow | 236 | 213 | 172 | 1,0 |
| avocado (avocado) | green | 88 | 122 | 46 | 0,9 | leeuw (lion) | brown | 232 | 210 | 137 | 0,8 |
| banaan (banana) | yellow | 239 | 216 | 84 | 0,9 | meloen (melon) | green | 44 | 87 | 18 | 1,0 |
| band (tire) | black | 0 | 0 | 0 | 0,8 | neus (nose) | rose | 228 | 176 | 146 | 1,0 |
| basketbal (basketball) | orange | 255 | 119 | 0 | 0,9 | pasta (pasta) | yellow | 242 | 229 | 191 | 1,0 |
| beer (bear) | brown | 108 | 66 | 54 | 1,0 | peer (pear) | green | 154 | 185 | 56 | 0,9 |
| bij (bee) | yellow | 244 | 215 | 118 | 1,0 | pinda (peanut) | brown | 178 | 145 | 38 | 0,9 |
| blad (leaf) | green | 113 | 130 | 28 | 1,0 | plant (plant) | green | 72 | 129 | 34 | 1,0 |
| bloemkool (cauliflower) | white | 220 | 201 | 122 | 0,9 | pompoen (pumpkin) | orange | 255 | 139 | 45 | 1,0 |
| broccoli (broccoli) | green | 102 | 153 | 52 | 1,0 | prei (leek) | green | 134 | 176 | 52 | 0,9 |
| brood (bread) | brown | 152 | 91 | 33 | 1,0 | schildpad (tortoise) | green | 65 | 73 | 27 | 1,0 |
| cactus (cactus) | green | 113 | 123 | 86 | 1,0 | sinaasappel (orange) | orange | 246 | 157 | 39 | 0,9 |
| champignon (mushroom) | white | 206 | 190 | 154 | 0,9 | sla (lettuce) | green | 127 | 166 | 57 | 1,0 |
| citroen (lemon) | yellow | 255 | 247 | 0 | 1,0 | sprinkhaan (grasshopper) | green | 108 | 235 | 108 | 1,0 |
| croissant (croissant) | brown | 248 | 191 | 112 | 0,9 | tak (branch) | brown | 121 | 93 | 71 | 0,9 |
| denneappel (pinecone) | brown | 163 | 108 | 51 | 1,0 | tand (tooth) | brown | 250 | 249 | 217 | 1,0 |
| dinosaurus (dinosaur) | green | 136 | 147 | 79 | 0,9 | tank (tank) | green | 76 | 90 | 64 | 0,9 |
| ei (egg) | white | 255 | 255 | 255 | 1,0 | tennisbal (tennis ball) | green | 200 | 236 | 74 | 0,9 |
| elleboog (elbow) | rose | 250 | 232 | 192 | 1,0 | tomaat (tomato) | red | 208 | 49 | 27 | 1,0 |
| flamingo (flamingo) | rose | 242 | 117 | 157 | 1,0 | ui (onion) | brown | 220 | 158 | 71 | 1,0 |
| framboos (raspberry) | red | 210 | 28 | 25 | 1,0 | verrekijker (binoculars) | black | 0 | 0 | 0 | 0,9 |
| granaatappel (pomegranate) | red | 201 | 8 | 38 | 0,9 | viool (violin) | brown | 170 | 84 | 27 | 1,0 |
| kaas (cheese) | yellow | 252 | 250 | 179 | 1,0 | voet (foot) | rose | 228 | 176 | 146 | 1,0 |
| kikker (frog) | green | 108 | 147 | 0 | 1,0 | vos (fox) | orange | 211 | 119 | 56 | 0,9 |
| kiwi (kiwi) | green | 156 | 183 | 74 | 1,0 | wereldbol (globe) | blue | 141 | 185 | 170 | 0,8 |
| knoflook (garlic) | white | 250 | 239 | 205 | 1,0 | witlof (chicory) | green | 193 | 227 | 93 | 1,0 |
| koffiebonen (coffee beans) | brown | 92 | 69 | 0 | 1,0 | zand (sand) | brown | 179 | 157 | 100 | 0,7 |

**Supplementary Table 2.** List of low color-diagnostic items used in Experiments 1 and 2 (translations in parentheses).

| **Low color-diagnostic items** | | | |
| --- | --- | --- | --- |
| aansteker (lighter) | hamer (hammer) | microscoop (microscope) | silo (silo) |
| armband (bracelet) | handdoek (towel) | nagellak (nail polish) | sok (sock) |
| auto (car) | handschoen (glove) | nietmachine (stapler) | speen (pacifier) |
| bal (ball) | hoed (hat) | oorbellen (earrings) | stoel (chair) |
| ballon (balloon) | kaars (candle) | ovenwant (oven mitt) | strik (bow) |
| biljartbal (billiard ball) | kam (comb) | paraplu (umbrella) | tandenborstel (toothbrush) |
| bloem (flower) | kano (canoe) | pen (pen) | telefoon (phone) |
| borstel (brush) | kast (closet) | pil (pill) | vergiet (colander) |
| elastiek (elastic) | klok (clock) | portemonnee (wallet) | veter (lace) |
| emmer (bucket) | knoop (button) | puntenslijper (pencil sharpener) | vlinder (butterfly) |
| fiets (bike) | kruiwagen (wheelbarrow) | riem (belt) | waaier (fan) |
| fluitje (whistle) | kussen (cushion) | rok (skirt) | washandje (washcloth) |
| föhn (hairdryer) | kwast (brush) | schaar (scissors) | wasknijper (clothespin) |
| gitaar (guitar) | lamp (lamp) | schoen (shoe) | wol (wool) |
| haarband (headband) | map (folder) | schroevendraaier (screwdriver) | zeppelin (zeppelin) |
